# *“I have come to remove it because of heavy bleeding”*: a mixed-methods study on early contraceptive implant removal and the underlying factors in eastern Uganda

**DOI:** 10.1186/s40834-024-00279-7

**Published:** 2024-04-16

**Authors:** Janet Abiyo, Rose Chalo Nabirye, Brendah Nambozo, David Mukunya, Ritah Nantale, Faith Oguttu, Solomon Wani, Milton W. Musaba, Josephine Tumuhamye, Joshua Epuitai

**Affiliations:** 1 Department of Nursing, Faculty of Health Sciences, Busitema University, Mbale, P.0 Box 1460, Uganda; 2 Department of Community and Public Health, Faculty of Health Sciences, Busitema University, Mbale, P.0 Box 1460, Uganda; 3 Department of Obstetrics and Gynaecology, Faculty of Health Sciences, Busitema University, Mbale, P.0 Box 1460, Uganda; 4 Busitema University Centre of Excellency for Maternal Reproductive and Child Health, Mbale, Uganda; 5 Makerere University Hospital, Makerere University Kampala, Kampala, P.O.BOX 7062, Uganda

**Keywords:** Contraceptive implants, Early implant removal, Long-acting contraceptives (LARCs), Mixed methods, Uganda, Family planning

## Abstract

**Background:**

Early contraceptive implant removal without intentions to conceive predisposes women to unintended pregnancies.. Some of the unintended pregnancies end in unsafe abortions which further increases the risk of maternal mortality and morbidity. Therefore, we assessed the proportion of women who had early contraceptive implant removal. We also explored the reasons for early contraceptive implant removalamong women at Mbale Regional Referral Hospital in eastern Uganda.

**Methods:**

We conducted a sequential explanatory mixed methods study at Mbale Regional Referral Hospital between November 2022 to December 2022. For quantitative data, we performed a secondary analysis on data extracted from the integrated family planning registers. We used systematic random sampling to select 600 clients’ serial numbers from the registers. The outcome variable was early contraceptive implant removal defined as removal of the implant by the woman before 18 months from the time of insertion. For qualitative data, we conducted 11 in-depth interviews among women who had come for contraceptive implant removal at the family planning clinic. We also conducted two key informant interviews with midwives working at the family planning unit. Quantitative data were analysed using Stata version 14.0 (Stata Corp LLC, College Station, Texas, USA) while qualitative data were analysed by thematic content analysis.

**Results:**

In this study, 15% (91/600) of the women discontinued contraceptive implants within 12 months, 29% (175/600) within 18 months, 38% (230/600) within 24 months and 40% (240/600) within 36 months of insertion. Among the women who discontinued contraceptive implant use, only 6.7% (40/600) switched to another family planning method. Out of the 175 women who removed contraceptive implants early, side effects 61.1% (107/175) desire to conceive 53.1% ( 93/175),, and gender-based violence 8.6% (15/175) were the major reasons for removal. From the qualitative interviews, the major reasons for early contraceptive implant removal were side effects such as heavy menstrualbleeding.

**Conclusion:**

A third of women discontinued contraceptive implant use within 18 months. Addressing concerns regarding side effects and male partner disapproval of modern contraceptives may improve continued use of implants.

## Background

The uptake of modern contraceptives has increased from 74% in 2000 to 77% in 2020 [1]. In sub-Saharan Africa, the unmet need for family planning is estimated at 23% (95% CI: 20.9–25.0) [2, 3]. In Uganda, the percentage of women aged 15–49 currently using a contraceptive method is 38% married women and 43% for sexually active unmarried women [4]. The unmet need for family planning is 22% of the total demand for family planning [4]. Currently, six per cent of married women use implants in Uganda [5]. Contraceptive implants are small flexible rods that are impregnated with progestin hormones to provide contraception [6]. In Uganda, contraceptive implants are available in the form of Implanon NXT® which is one rod containing 68 mg of etonogestrel and provide contraception for three years [7]. Levoplant® and Jadelle® both contain two rods each consisting of 75 mg levonorgestrel where they are effective for four years and five years respectively [6]. The implants are long-acting contraceptives (LARC) which are highly effective, safe for almost all women of reproductive age and are cost-effective [6]. The insertion and removal of contraceptive implants is a relatively easy procedures and the method is not dependent on user compliance [6].

The rights-based approaches to family planning emphasize that women are free to discontinue a family planning method at any time and for any reason [8]. Discontinuation maybe related to contraceptive failure (e.g., pregnancy), no continued need for contraception (e.g., menopause) or method related reasons (e.g., side effects) [7]. Women who discontinue may become pregnant, switch to use another method or completely abandon contraception use [7]. Complete discontinuation of contraceptives while still in need of contraception predisposes women to an increased risk of unintended pregnancy, closely spaced pregnancies, and the associated complications unsafe abortion, and high-risk pregnancies [7, 9, 10]. Globally, out of the 70% of women who discontinued contraceptive implant use while still in need of contraception, 15–20% were at risk of pregnancy three months after discontinuation [7].

Despite the benefits of contraceptive implants and the increase in the use of modern contraception, early contraceptive implant removal rates remain high in low and middle-income countries [11]. Globally, about 9% of women discontinue contraceptive implants within a year of insertion [7]. In East Africa, one in three couples are likely to discontinue the use of contraception within a year [12]. In Uganda, 21% of users of contraceptive implants discontinue within a year [13]. A study done in central Uganda reported a discontinuation rate of 42% of contraceptive implants within 18 months of insertion [14]. However, the study was small (about 200 participants) and did not explore the experiences of participants regarding early contraceptive implant removal. The government of Uganda recognizes the role of family planning in achieving the Uganda vision 2040 target of reducing the population growth rate from 3.2 to 2.4% to reap the demographic dividend [15]. The government is committed to the 2030 family planning target of increasing the modern contraceptive prevalence rate (mCPR) for all women from 30.4% in 2020 to 39.6% by 2025 and reducing unmet needs from 17% in 2020 to 15% by 2025 [15]. Understanding the proportion and reasons for early contraceptive implant removal among women is key to guiding interventions towards attaining the family planning goals of the country as well as the sustainable development goal 3. To the best of our knowledge, limited studies have been conducted in our setting, a region with one of the highest fertility rates and lower rates of contraceptive implant use. Therefore, we assessed the proportion and reasons for early contraceptive removal among women in eastern Uganda.

## Methods and materials

### Study design

We conducted a sequential explanatory mixed methods study [16]. For the quantitative phase, we performed a secondary analysis of data from the integrated family planning registers for the past three years from October 2019 to October 2022 to assess the proportion of women who had removed contraceptive implants early. For the qualitative phase, we borrowed upon the phenomenological strategy of inquiry which focused on participant’s experiences and reasons for early contraceptive implant removal [16].

### Study setting

We conducted this study at the family planning clinic of Mbale Regional Referral Hospital (MRRH) in Eastern Uganda which is approximately 230 km east of the capital Kampala. The hospital serves as a referral centre for four district hospitals and ten health sub-districts in and around Mount Elgon zone. The hospital is a government facility with a bed capacity of 415 and offers specialized healthcare services including family planning services to a catchment area of 4.5 million people.

### Study population

We reviewed records of all women of reproductive age between 15 and 49 years of age who received family planning services and health workers at the family planning clinic of Mbale Regional Referral Hospital. Healthcare providers who were working in the family planning clinic in the hospital were included, while women who were seeking contraceptive implant removal were also included in the qualitative study.

### Eligibility

#### Inclusion criteria

We analyzed data of all women of reproductive age who had contraceptive implant removal between October 2019 and October 2022 but with records of the date of insertion of the contraceptive implant. In the qualitative interviews which required obtaining consent from individuals, we included only women aged 18 years and above who came for implant removal at the family planning clinic during the study period.

#### Exclusion criteria

Data from all women of reproductive age with missing records in the register of either implant insertion or removal. For the qualitative interviews, we excluded women who came for implant removal but declined to provide informed consent.

### Sampling technique

#### Quantitative

We systematically sampled clients’ serial numbers in the registers and selected study participants. We obtained a sampling interval by dividing 1249 (total number of women who had removed implants in the past three years) by 594(study sample size) which equalled two [17]. The initial client’s serial number was randomly selected from the first two records in the register [17]. The next client’s serial number was obtained by adding two to the initial number, and the process continued systematically [17].

#### Qualitative

We purposively sampled women to participate in the in-depth interviews and health workers for the key informant interviews. Only participants who came for implant removal during the study period were interviewed. We selected nurses and midwives who provide family planning services to women in the family planning unit to participate in the key informant interviews. These health workers provide a range of services like pre and post-insertion counselling, provision of family planning methods, removal of some methods like implants and IUDs and management of clients with side effects.

### Sample size estimation

#### Quantitative sample size

We used the Cochran formula to estimate the number of participants in the study [18]. We assumed a 45% proportion of early contraceptive implant removal based on the Uganda Demographics and Health Survey (2016) [19] and a precision of 4%. This gave us a total sample size of 594 participants,

#### Qualitative sample size

We used the principle of data saturation to estimate the sample size [17].

### Data collection

Quantitative data: We collected quantitative data from the integrated family planning registers using a data extraction tool which was developed from literature about early contraceptive implant removal for collecting quantitative data [2, 11, 19, 20]. We identified all the names of women who had removed implants from the integrated family planning register and traced their dates of implant insertion from the implant consent books to estimate the period of implant use. The registers had data on age, residence, contraceptive method given or removed and reasons for removal. The reasons for removal of contraceptive implants were pre-specified in the integrated family planning registers. The reasons included side effects, gender-based violence, desire to conceive, due date, and desire to switch to another method, while the option of others allowed capture of information which was not in line with pre-specified categories. Routinely, clients who come to remove the contraceptive implants are asked to provide their main reason for removal which is captured in the registers by the midwife working in the family planning unit. We entered all data from the extraction tool into Excel spreadsheets, and cleaned and categorized it.

We collected qualitative data using an interview guide with open-ended questions. The in-depth interviews were conducted by JA, a nursing student in the final year of study. Interviews were conducted in English and the commonly spoken local languages. All interviews were audio recorded and later transcribed verbatim. The questions explored the experience of women with the use of contraceptive implants, pre-insertion counselling, duration of contraceptive implant use, reasons for removal and conception plan after removal of the contraceptive implants. We also conducted two key informant interviews among the health workers providing contraceptive implants to women at the family planning unit. The questions in the key informant guide explored reasons for early implant removal, pre-insertion counselling and experiences of women who removed the contraceptive implants early.

### Study variables

Our outcome variable was early contraceptive implant removal which was considered in the study as the removal of a contraceptive implant at 18 months or less after insertion. Early contraceptive implant removal was measured as a binary variable coded as ‘yes’ if the implant was removed before 18 months from the time of insertion and ‘no’ if removed after 18 months. The reasons for early removal included due for removal, change in fertility interest and gender-based violence. The due date referred to 3- or 5-year period when the implants were to be removed because its effectiveness would reduce after this period. The due date was captured in the hospital register as a period exceeding 36 months post-insertion.

### Data quality control

We pre-tested the data extraction tool at Mbale Regional Referral Hospital Family Planning Unit. The tool was cross-checked and compared with the available information in the family planning clients register. All information was checked for correctness of transcription as well as checking for incomplete responses.

### Data analysis

Quantitative data were analysed using Stata version 14.0 (Stata Corp LLC, College Station, Texas, United States of America). We used percentages and frequencies to describe discontinuation of contraceptive implants, while frequencies and percentages were used to summarise the reasons of early discontinuation of contraceptive implants contraceptive implant. We summarised categorical variables using frequencies and percentages while continuous variables using means and standard deviations.

For qualitative data, we transcribed all recordings of in-depth and key informant interviews verbatim. All transcripts were reviewed against the recorded interviews for accuracy. We analysed the transcripts “Thematic Analysis” method. We analysed the transcripts “Thematic Analysis” method. Two study team members (AJ and JE) independently coded the interviews and later met and agreed on the coding framework, using a combination of deductive and inductive approaches [21]. All interviews were then coded and the coding framework was developed iteratively to add new emerging concepts. After completing coding, the study team (JA, JE, BN, DM, RN, FO, SW) met and grouped the codes into themes and subthemes to explain the experiences of women with contraceptive implant use and the reasons for early removal.

### Ethical consideration

We obtained ethical approval from Mbale Regional Referral Hospital Research and Ethics Committee (MRRH-REC-220). We also sought administrative clearance from Mbale Regional Referral Hospital to retrieve data from the integrated family planning registers. The dataset was anonymised with no client identifiers like names or addresses. The address captured was generic as it referred to the district of origin or residence. For the qualitative interviews, we obtained written informed consent from the study participants before enrolling them into the study. The transcripts do not contain the names or addresses of the study participants, while codes have been used to describe the participants. The qualitative and quantitative data was kept confidential, on a password protected computer that was accessible only to the study team.

## Results

### Study description

**Fig. 1:**
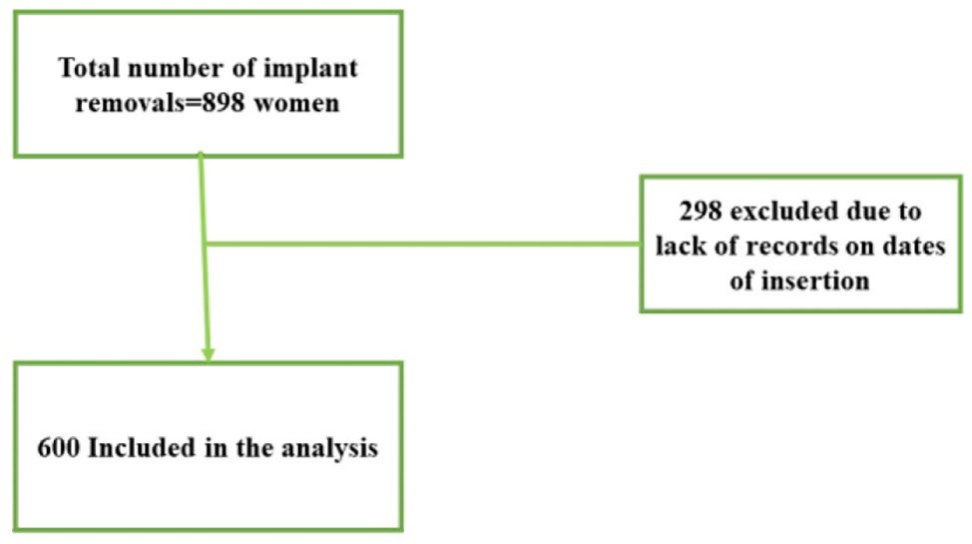
Sample included in the study

Out of the 898 records, 298 were excluded from the study because of missing data (Fig. 1). Data of 600 women were complete and was included in the final analysis of the study. This represented 67% of all the available hospital records in the study period.

### Socio-demographics characteristics of study participants

We collected data from a total of 600 client’s serial numbers. Among the socio-demographic characteristics, only age and address were captured in the integrated family planning registers. The mean age was 31.0 years with a standard deviation of 7.1 years. The majority of the participants 286/600 (47.7%) were aged 25–35 years and most 516/600(86%) were from Mbale.

### Proportion of early contraceptive implant removal among analysis sample at Mbale Regional Referral Hospital

**Fig. 2:**
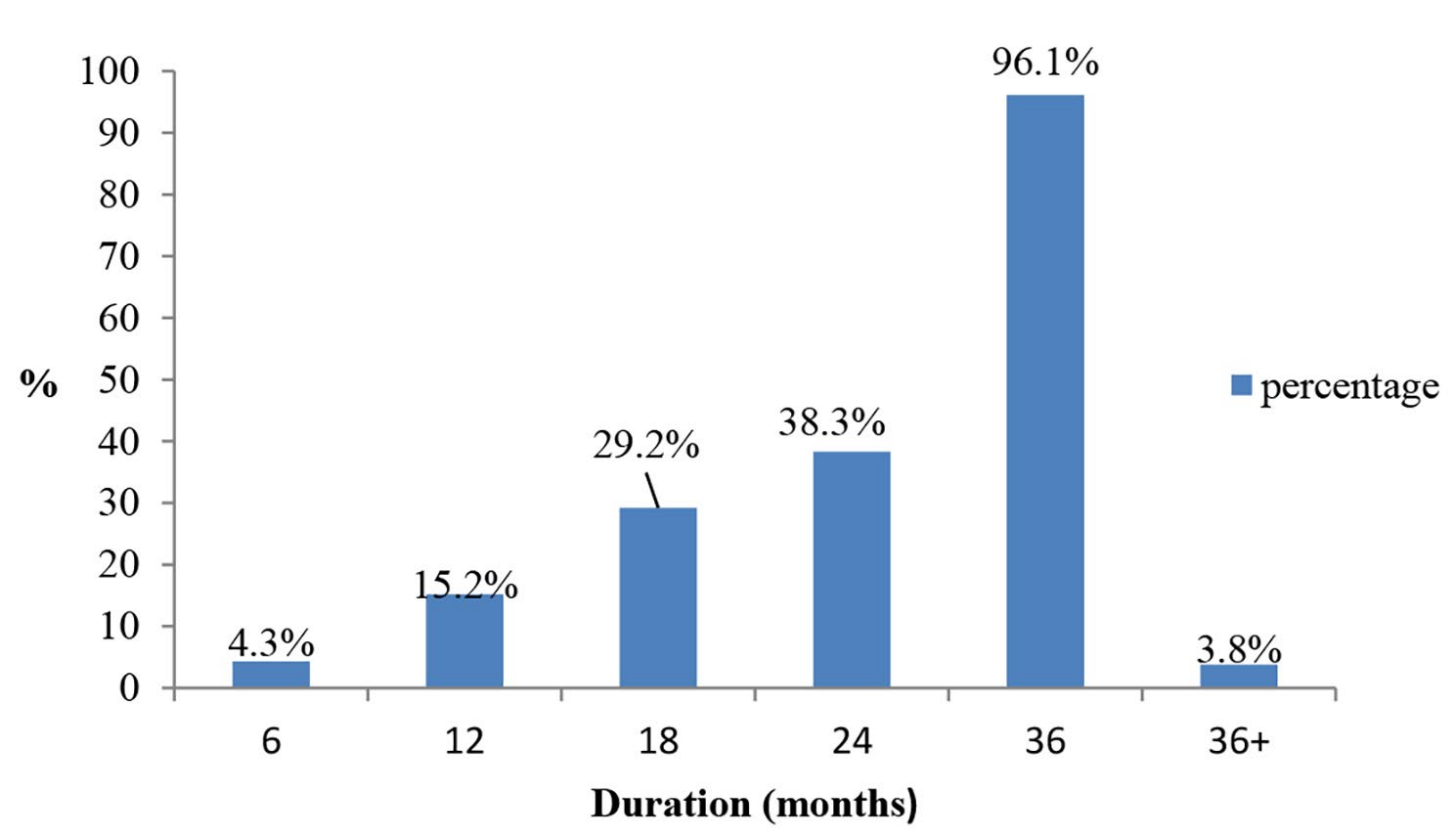
Proportion of contraceptive implant removal in MRRH

The proportion of early contraceptive implant removal was 29.2% (175/600) using a cut-off of 18 months (Fig. 2). The mean duration of use with the contraceptive implant was 28.4 months with a standard deviation of 12.6 months.

### Reasons for contraceptive discontinuation

Among the women who discontinued contraceptive implants early before 18 months, over half 51.4% (90/175) were due to side effects (Table 1). Nearly one-third 30.3% (53/175) discontinued contraceptive implant use early because of a change in fertility desire and 8.6% (15/175) due to gender based violence. The reasons for removal of the contraceptive implant before the 36 months when the implant use expired were side effects (44.6%, 107/240), desire to conceive (38.7%, 93/240) and gender-based violence (6.3%, 15/240).

**Table 1:** Reasons for contraceptive implant removal

Variables	Frequency (%)
Overall discontinuation *n* = 600	
After 36 months Before 36 months	360 (60.0) 240 (40.0)
Reasons for removal before 36 months *n* = 240 Side effects Change in fertility interest Gender based violence Others	**n (%)** 107(44.6) 93(38.6) 15(6.3) 25(10.4)
Reasons for early removal before 18 months *n* = 175 Side effects Change in fertility interest Gender based violence Others^*^	**n (%)** 90(51.4) 53(30.3) 15(8.6) 17(9.7)

*Others = unknown reasons

### Proportion of women who switched to other contraceptive methods after removing contraceptive implants

**Fig. 3:**
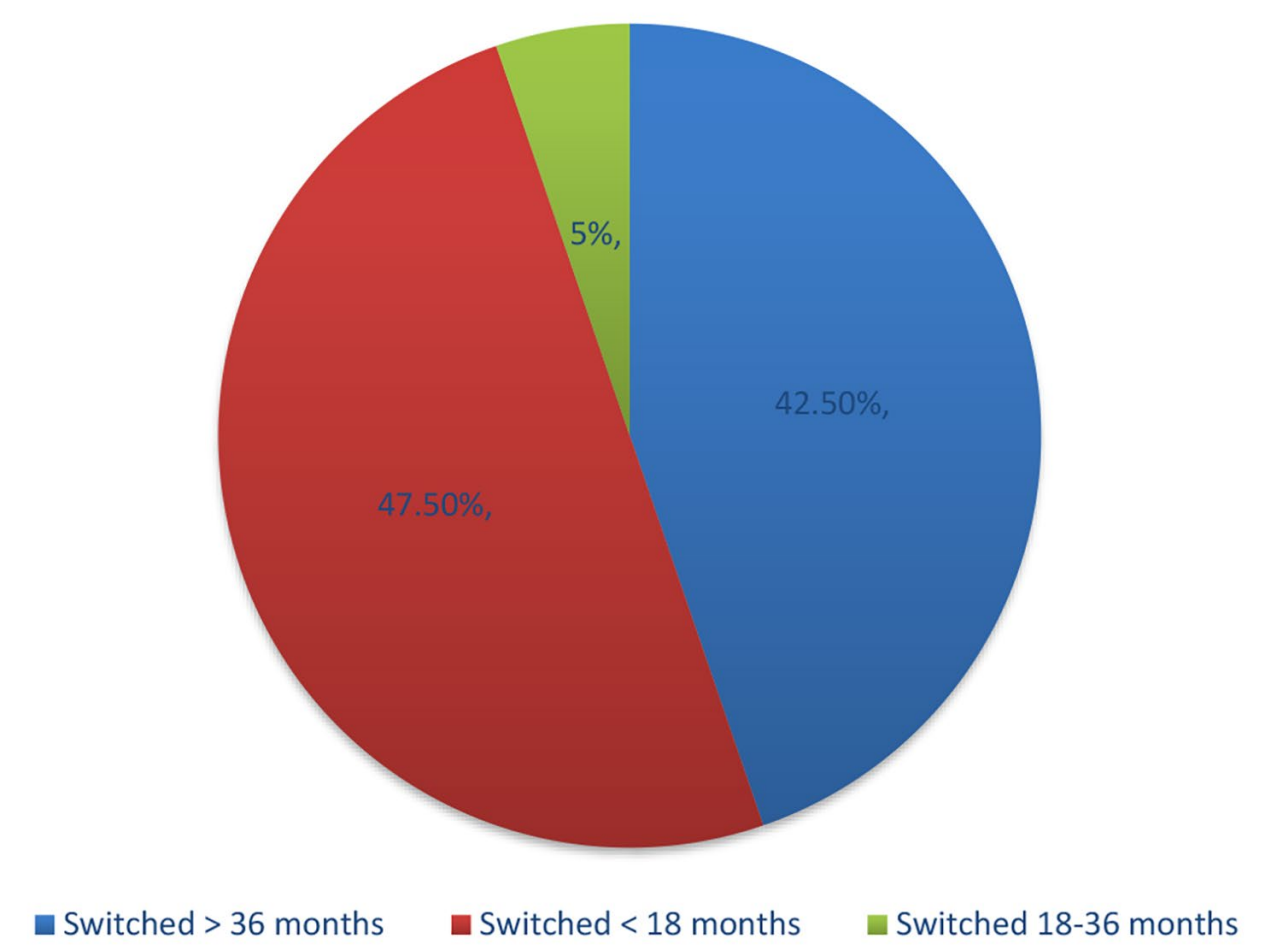
Proportion of women who switched to another method

A total of 40/600 (6.7%) changed to another family planning methods after contraceptive implant removal. Among women who changed to another family planning method, 42% (*n* = 17) were because of the due / expiry date of the implant at 36 months, while 47% (*n* = 21) of women removed implant early before 18 months from insertion (Fig. 3). Experiencing side effects (17/21, 81%) and gender-based violence (4/21, 19%) were the main reasons from removal implants before 18 months.

### Qualitative results

#### Socio-demographic characteristics of participants

A total of eleven in-depth interviews (nine women and two health workers) were conducted to explore the reasons for early contraceptive implant removal. The participants were aged between 22 and 49 years with a range of 1–6 children. Seven participants were married, four were business-women and six had attained a primary level of education. The midwives were aged 42 and 47 years and were married.

We grouped the results into four major themes: side effects of contraceptive implants, partner opposition, desire to have another baby, counselling, misconception and community opposition. Details are shown in table.

#### Side effects of contraceptive implants

Side effects of contraceptive implants were repeatedly mentioned a reason for early removal of contraceptive implants. The side effects included headache, dizziness, weakness, weight gain, weight loss and heavy menstrual bleeding. Some women further reported that heavy menstrual flow interfered with their sexual life as they could not get intimate with their partners. One participant told us:

*“He thinks I am cheating on him that is why I do not want to give him (sex). At times he can try to force me, finds blood (menstrual blood) and after he says he is sorry.” (A discontinuer at 16 months due to side effect, IDI2)*.

Another participant said:

*“I have come to remove it because of heavy bleeding… my partner can be expecting something good (sex) and I tell him stories…. every time he feels like trying the condition worsens.” (A discontinuer at 12 months due to side effects, IDI8)*.

Heavy menstrual bleeding exposed some women to sexual violence by their partners because they are unable to be intimate with their partners.

Key informants observed that women with side effects were not willing to be counselled but instead were only open for the implant to be removed.

*“When they come back with side effects, most of them just come for removal, but just like I*.

*said you talk to them and manage side effects” (Midwife, KII2).*

#### Inadequate pre-insertion counselling about family planning

Experiencing side effects of contraceptive implants without receiving pre-insertion counselling increased the likelihood of some women removing the implants after a short period of use. Some participants reported not having been taught about the side effects of implants. One of the health workers mentioned that women who were not counselled about the side effects of the contraceptive implants discontinued use of implant immediately after experiencing the side effects.

*“No, they did not tell me anything (pre-insertion counselling), what I remember is that I went with my mother and by that time I knew nothing about family planning. My mother talked with the nurses around and I was taken to a room where the implant was inserted without telling anything. (A discontinuer at 16 months due to side effects and partner opposition, IDI 4)*

### Partner/community disapproval and misconception

Women and health workers reported partner opposition as one of the reasons for early contraceptive implant removal. Participants, who did not discuss with their male partners before insertion, were coerced to remove the implants as soon as their partners discovered. In addition, married women who experienced heavy menstrual flow as a side effect were forced by their partners to remove the contraceptive implant early even though they had endured the side effects like heavy bleeding. One participant told us:

*“I did not tell him when I was going to insert it, I just went and inserted it without his knowledge…today he told me to go and they remove that thing (contraceptive implant)” (a discontinuer at 18 months due to pressure from partner, IDI7).*

She also added:

*“I had endured heavy bleeding but the issue of my husband abusing me and quarrelling all the time, I no longer have peace at home. Every time he comes back home, he starts quarrelling and he is always asking me if I went and had the implant removed. He has even escorted me today to make sure that I have this thing (implant) removed.” (A discontinuer at 18 months due to pressure from partner, IDI7)*.

Another participant said:

*“It has even brought us separation and quarrels. He calls me a prostitute…. Please don’t convince me to remain with this implant because I am not going back with it…. My husband told me not to go back with it. He always says if it was possible to pull out this thing (implant), he would have pulled it out a long time ago.” (A discontinuer at 16 months due to pressure from partner, IDI3).*

Misconceptions in the community about contraceptive implants were also reported as a reason for early implant removal. Health workers reported that communities associate any abnormality with family planning. One of the health workers also reported that the myths heard by women in the community after insertion scare them. One health worker told us:

*“Some of them say when implants are inserted, they disappear in the body. Others say it will cause cancer while others say you will deliver children without limbs. They associate any abnormality with family planning.” (Midwife, KII 1)*.

Another health worker said:

*“Community members tell them you have decided to use that method but that method is not good, it will get lost in your body, and it will go to the head, the stomach and the back. These things really scare the clients then they come back asking for removal.” (Midwife, KII2).*

The community plays a role in spreading misinformation about contraceptive implants, and as a result, scares women who are using contraceptive implants. Fear of the negative implications causes these women to remove the contraceptive implants before the due date.

#### Desire to have another baby

Some participants reported the need to have another baby as the reason for early contraceptive implant removal. They stated that they used family planning until their babies reached a certain age before they got another one. One participant told us:

*“I just feel like I need to remove it (the contraceptive implant) because my girl is already five years and I need another baby.” (A discontinuer at 25months due desire to have another baby IDI1)*.

*“I need another baby because my last born has grown…. I want to first give birth to four more children because I want to make a total of ten children.” (A discontinuer at 12 months due to desire to have more children, IDI8)*.

The desire to have another baby is a legitimate reason to have a contraceptive implant removed. These women were prepared to conceive therefore had to remove the contraceptive implants.

## Discussion

This study assessed the proportion of early contraceptive implant removal and explored the reasons for early removal. A third of the women removed the contraceptive implants before 18 months. The major reasons for early contraceptive removal were side effects, partner opposition and a change in fertility interest.

### The proportion of women who had an early contraceptive implant removal

Nearly one-third of participants (29%) in our study removed contraceptive implants early. This could imply that some women are likely to have unintended pregnancies especially if their reason for early contraceptive implant removal was not a desire to have another child [22, 23]. For instance, in our study, almost 70% of women removed contraceptive implants before 18 months for reasons other than the desire to conceive, which could imply that they are at risk of unwanted pregnancy and its associated complications of unsafe abortion, maternal morbidity and mortality. According to a recent report by the Lancet, sixty-one per cent of unintended pregnancies end in unsafe abortions [24] which greatly contribute to maternal mortality [25]. The proportion of women who removed contraceptives early in our study was higher than that found elsewhere. For instance, Nega et al., in Ethiopia found a 23.2% rate of early removal of contraceptive implants [26] while Akimali et al., found a rate of 20% in the Democratic Republic of Congo [27] and a study in Thailand found a rate of 8.9% [28]. A possible explanation could be a lack of effective pre-insertion counselling about the side effects of contraceptive implants. From our in-depth interviews some women reported not receiving any counselling before receiving the contraceptive implants. Such women are unaware of the possible side effects of contraceptive implants and will most likely opt for an early removal in case they experience any side effects such as menstrual irregularities. On the other hand, our findings are lower than those from other studies for example a study in central Uganda found an early discontinuation rate of 42% [14]. Other studies in Ethiopia found a rate of 49.3% [29], 38% [30] and 65% [10]. This difference may be attributed to socio-cultural differences among the study settings.

### Reasons for early contraceptive implant removal

Our study found side effects to be the primary reason for early discontinuation of contraceptive implants. Our findings are consistent with other studies done in Kampala, Uganda [14], Ethiopia [30], and Thailand [28] which reported side effects as the major reason for early discontinuation. Side effects like excessive menstrual bleeding may interfere with a woman’s domestic, work and social life [31]. For example, from the qualitative interviews, while some women tolerated the side effects like heavy menstrual flow, they were constantly coerced and abused by their partners through psychological pressure, sexual and physical violence [32, 33]. Previous studies have cited the critical role of gender-based violence and reproductive coercion with reduced access to and use of modern contraceptives [34, 35]. Reproductive coercion and abuse may manifest in the form of contraception sabotage and interference with the use of contraception including forceful attempts to remove contraceptive implants [32]. Women respond to reproductive coercion by opting for modern contraceptives that are concealable which may result in partner abuse once the partner notices the practice of contraceptive use [32, 33]. Partner opposition could be attributed to fear of side effects, differences in fertility interests and negative perceptions of men towards family planning. In addition, partner opposition may be related to the patriarchal nature of the societies in which these studies were conducted. Men are considered key decision makers in the family and failure of the women to involve them before getting a family planning service might result in opposition as it undermines their authority. There is also a possibility that many men are not involved in pre-insertion counselling before their partners are given contraceptive implants and in the event of side effects, they pressure them to discontinue the method early. Empowering women, community engagement approaches and health education of communities regarding family planning may help reduce male partner opposition in the use of modern contraceptives including intimate partner violence from use of modern contraceptives. In addition, the poor quality of counselling before insertion of the contraceptive implants could also affect how a woman responds to the possible side effects and thereby contribute to early removal. Policy makers of family planning services should explore the possibility of providing phone numbers of health facilities which women could use to call to consult, while community healthcare workers could follow-up women in the post-insertion period to identify, assess and manage those with side effects. Furthermore, there is a need for effective pre-insertion counselling to women and their partners to minimise early contraceptive implant removal. Women who receive clear information might tolerate side effects and are more likely to continue with the method [26].

Myths and misconceptions were also reported as reasons for early contraceptive implant removal. Female peers and family are key sources of information regarding a woman’s choice of contraceptive method. Beliefs, myths and misconceptions may arise from personal experiences or information from other people. These beliefs act as psychosocial barriers to women either accessing or continuing the use of a contraceptive method especially when one is negatively judged for using contraceptives by the community [36]. Therefore, there is a need to investigate the individual beliefs/misconceptions among contraceptive users and nonusers to develop strategies to address these barriers at the community level. Myths and misconceptions in the community should be resolved through different education modalities on contraceptive implants [26]. Effective counselling of all clients before implant insertion also empowers women with the right information and will most likely not be overwhelmed by the misinformation therefore reduce early contraceptive implant removal.

Gaps in the quality of pre-insertion counselling was cited by some participants and health providers as a reason for early removal. Women who were not counselled about the side effects of contraceptive implants were more likely to have an early contraceptive implant removal. This finding was consistent with studies conducted in Ethiopia [26, 37]. When women are thoroughly informed about the method during pre-insertion counselling, this encourages realistic expectations and could minimise early removal of contraceptive implants. These women might tolerate minor side effects and thus are more likely to continue with the method [26]. Therefore, effective counselling of all clients before implant insertion will reduce early contraceptive implant removal.

### Study limitations

Firstly, our findings might not be generalized to the general population including the rural community because the study was conducted in an urban health facility and might not be representative of the rural population. Secondly, the qualitative findings might not reflect the general population’s perceived reasons for early discontinuation since we conducted a few in-depth interviews and two key informant interviews. Lastly, this study did not assess for some important socio-demographic factors like marital status, parity and level of education which were not captured in the integrated family planning registers.

## Conclusion

Nearly one-third of women discontinued contraceptive implant use early because of mostly side effects of the method and gender-based violence. In the qualitative interview, women came for contraceptive implant removal because of side effects, partner and community opposition, myths and misconceptions, and desire to conceive. Improving the quality of counselling before insertion of contraceptive implants is critical in promoting sustained use of contraceptive implant especially for women in need of contraception and who do not wish to conceive. Public health efforts should not just be limited at promoting uptake of contraceptive implants but should extend to target current users of contraceptive implants to provide further health information to dispel misconceptions, empower women in use of contraceptives, involve male partners and manage the existing side effects of the contraceptive method. Lastly, educational interventions targeting communities should be explored to empower them with information on contraceptive methods and dispel myths and misconceptions.

## Data Availability

Additional data and materials can be accessed on reasonable request from the corresponding author.
